# 6-(2-Hydroxy­phen­yl)-5,6-dihydro­benzimidazolo[1,2-*c*]quinazolin-12-ium bromide ethanol solvate

**DOI:** 10.1107/S1600536809032899

**Published:** 2009-08-22

**Authors:** Irvin Booysen, Thomas I. A. Gerber, Eric Hosten, Peter Mayer

**Affiliations:** a Department of Chemistry, Nelson Mandela Metropolitan University, 6031 Port Elizabeth, South Africa; b Department of Chemistry, Ludiwig-Maximilians University, D-81377 München, Germany

## Abstract

In the title compound, C_20_H_16_N_3_O^+^·Br^−^·C_2_H_6_O, the phenol ring forms dihedral angles of 84.5 (1) and 89.3 (1)° with the benzimidazole system and the quinazoline benzene ring, respectively. The two N—H groups act as donors in hydrogen bonds with the bromide ion as acceptor, leading to infinite eight-membered chains along [100]. According to graph-set theory the descriptor on the binary level is *C*_2_^1^(8).  O—H⋯O and O—H⋯Br hydrogen bonds also occur.

## Related literature

For the synthesis of quinazolines, see: Kubicova *et al.* (2003 ▶); Niementowski (1895 ▶). For related literature, see: Cuny *et al.* (1980 ▶); Williamson (1957 ▶). For graph-set analysis, see: Bernstein *et al.* (1995 ▶); Etter *et al.* (1990 ▶).
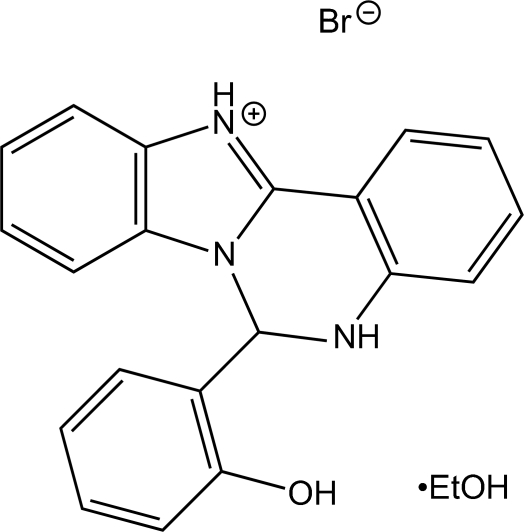


## Experimental

### 

#### Crystal data

C_20_H_16_N_3_O^+^·Br^−^·C_2_H_6_O
*M* *_r_* = 440.33
Triclinic, 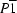

*a* = 9.3438 (5) Å
*b* = 10.0736 (5) Å
*c* = 10.8452 (5) Å
α = 86.832 (4)°
β = 77.203 (4)°
γ = 84.674 (4)°
*V* = 990.53 (9) Å^3^
*Z* = 2
Mo *K*α radiation
μ = 2.10 mm^−1^
*T* = 200 K
0.28 × 0.24 × 0.05 mm

#### Data collection

Oxford XCalibur diffractometer
Absorption correction: multi-scan (*CrysAlis RED*; Oxford Diffraction, 2006 ▶) *T*_min_ = 0.783, *T*_max_ = 1.000 (expected range = 0.705–0.900)
7695 measured reflections
4004 independent reflections
2664 reflections with *I* > 2σ(*I*)
*R*_int_ = 0.030

#### Refinement

*R*[*F* ^2^ > 2σ(*F* ^2^)] = 0.034
*wR*(*F* ^2^) = 0.057
*S* = 0.84
4004 reflections
256 parameters
H-atom parameters constrained
Δρ_max_ = 0.60 e Å^−3^
Δρ_min_ = −0.35 e Å^−3^

### 

Data collection: *CrysAlis CCD* (Oxford Diffraction, 2006 ▶); cell refinement: *CrysAlis RED* (Oxford Diffraction, 2006 ▶); data reduction: *CrysAlis RED*; program(s) used to solve structure: *SIR97* (Altomare *et al.*, 1999 ▶); program(s) used to refine structure: *SHELXL97* (Sheldrick, 2008 ▶); molecular graphics: *ORTEP-3 for Windows* (Farrugia, 1997 ▶); software used to prepare material for publication: *PARST* (Nardelli, 1995 ▶) and *publCIF* (Westrip, 2009 ▶1).

## Supplementary Material

Crystal structure: contains datablocks I, global. DOI: 10.1107/S1600536809032899/ng2630sup1.cif

Structure factors: contains datablocks I. DOI: 10.1107/S1600536809032899/ng2630Isup2.hkl

Additional supplementary materials:  crystallographic information; 3D view; checkCIF report

## Figures and Tables

**Table 1:** Hydrogen-bond geometry (Å, °)

*D*—H⋯*A*	*D*—H	H⋯*A*	*D*⋯*A*	*D*—H⋯*A*
O1—H1⋯O2^i^	0.84	1.82	2.657 (2)	175
N1—H71⋯Br1^ii^	0.88	2.61	3.3501 (17)	142
N3—H73⋯Br1^iii^	0.88	2.45	3.1956 (18)	143
O2—H2⋯Br1	0.84	2.41	3.2378 (17)	168

Symmetry codes: (i) 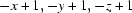; (ii) 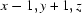; (iii) 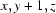.

**Fig. 1.**
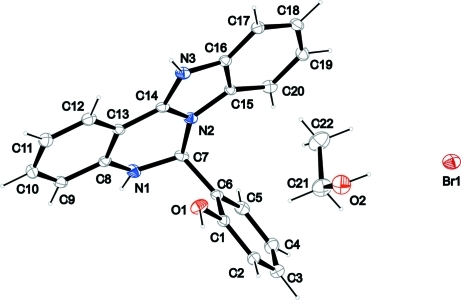
The molecular structure of the title compound (anisotropic displacement ellipsoids drawn at the 50% probablility level).

**Fig. 2.**
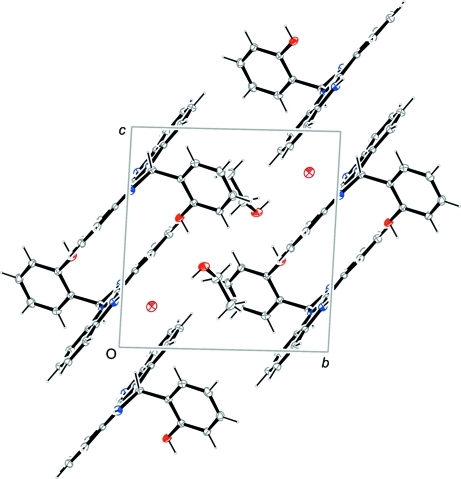
The packing of the title compound, viewed along [-100].

**Fig. 3.**
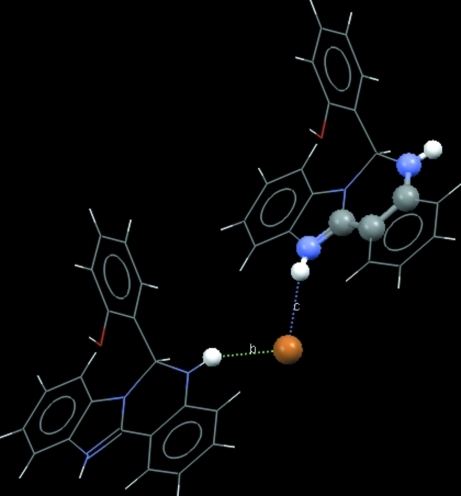
The hydrogen bonds leading to infinite 8-membered chains along [100]. b: N1–H71···Br1, c: N3–H73···Br1.

**Fig. 4.**
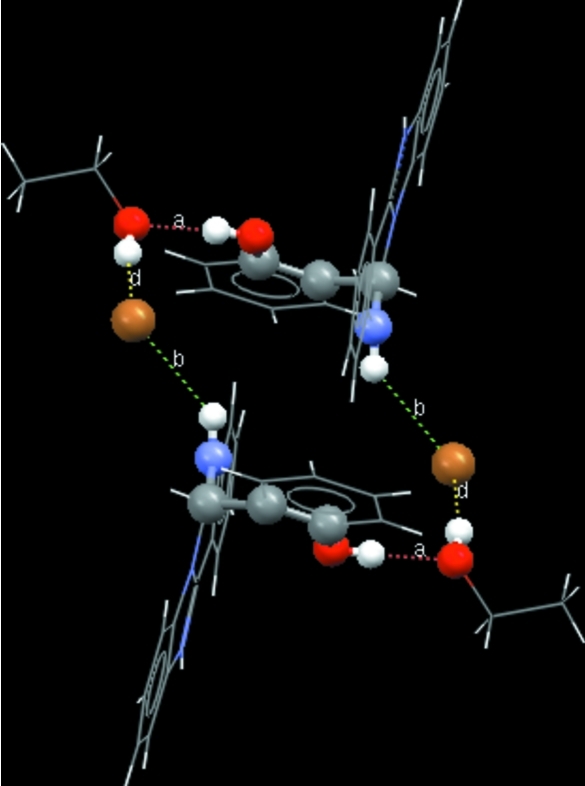
The hydrogen bonds leading to 20-membered rings. a: O1–H1···O2, b: N1–H71···Br1, d: O2–H2···Br1.

**Fig. 5.**
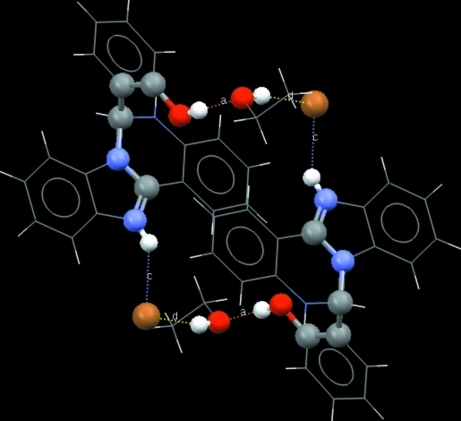
The hydrogen bonds leading to 24-membered rings. a: O1–H1···O2, c: N3–H73···Br1, d: O2–H2···Br1.

## supplementary crystallographic information

### Comment

In the present work the structure of 2-(tetrahydrobenzimidazolium[1,2-*c*]quinazolin-5-yl)phenol bromide has been determined to explore its suitability as a bidentate ligand for various metal ions. In the structure the quinazoline ring adopts a chair conformation: atoms C8, C13, C14, N1 and N2 are coplanar, with atom C7 from the plane by 0.178 Å (Figure 1). The orientation of the phenol ring is determined by a hydrogen-bond between the phenolic oxygen atom and the ethanolic oxygen atom. This ring makes dihedral angles of 84.5° and 89.3° with the benzimidazole and phenyl rings respectively. The ligand bond distances and angles show that N3–C14 is a localized double bond [1.337 (3) Å], with N2–C15 a single bond at 1.398 (3) Å. N3 is protonated, with the C14–N3–C16 bond angle equal to 109.30 (17)°. The N1–C7 bond length is 1.452 (3) Å, and the N1–C7–N2 bond angle [107.71 (16)°] illustrates the sp3 hybridization of C7.

The molecular packing of the title compound is shown in Figure 2. A feature of the structure is parallel stacking of the 5-membered ring N2—C14—N3—C16—C15 and the 6-membered ring C15—C16—C17—C18—C19—C20. These planes have an interplanar angle of 0.45 (11)° and an interplanar distance of 3.4118 (8) Å.

The two O–H groups and the two N–H groups act as donors in four different hydrogen bonds, three of them with bromide as acceptor and one of them with the ethanolic oxygen atom as acceptor. In terms of graph set analysis (Etter *et al.*, 1990; Bernstein *et al.*, 1995), three extended hydrogen bond patterns may be selected and characterized by graph set descriptors. 8-membered chains along [100] are formed by the two hydrogen bonds of the type N–H···Br (graph set descriptor C^1^_2_(8) on the binary level, Figure 3). A 20-membered ring and a 24-membered ring are formed by six hydrogen bonds within two formula units (Figures 4 and 5). The graph set descriptors *R*^4^_6_(20) and *R*^4^_6_(24), respectively, can be assigned on the ternary level.

### Experimental

All chemicals used (reagent grade) were commercially available. A mass of 0.0244 g (200 µmol) of 2-aminobenzaldehyde was dissolved in methanol (10 cm^3^), and 0.0418 g (200 µmol) of 2-(2-aminophenyl)-1-benzimidazole was added with stirring. After the mixture was heated under reflux for 30 min, a mass of 0.096 g (100 µmol) of *trans*-[ReOBr_3_(PPh_3_)_2_] was added, and heating was continued for a further 30 min. After cooling to room temperature, the solution was filtered and left to evaporate slowly at room temperature. After 2 days 0.063 g (72%) of colourless crystals, with the formulation [C_20_H_16_N_3_O]Br.C_2_H_6_O and suitable for X-ray analysis, were collected. *M*.p. 211°C. ^1^H NMR (300 MHz, d6-DMSO): 14.71 (1*H*, br s), 8.62 (1*H*,d), 8.58 (1*H*, d), 8.18 (1*H*, s), 8.04–8.09 (2*H*, m), 7.72–7.82 (3*H*, m), 7.68 (1*H*, dd), 7.42–7.48 (2*H*, m), 7.28 (1^H^, t), 7.19 (1^H^, t), 7.06 (1*H*, d), 3.42 (2*H*, q), 1.23 (3*H*, t). IR (KBr, cm^-1^): ν(OH) 3460w, ν(NH) 3275, ν(C=N) 1603 s.

### Refinement

The H atoms were positioned geometrically (C—H = 0.98 Å for CH_3_, 0.99 Å for CH_2_, 0.95 Å for CH, 0.84 Å for OH, 0.88 Å for NH) and treated as riding on their parent atoms [*U*_iso_(H) = 1.2*U*_eq_(C/N) for CH and NH, *U*_iso_(H) = 1.5*U*_eq_(C/O) for CH_3_ and OH].

### Crystal data

**Table:**

C_20_H_16_N_3_O^+^·Br^−^·C_2_H_6_O	*F*(000) = 452
*M**_r_* = 440.33	*D*_x_ = 1.476 (1) Mg m^−^^3^
Triclinic, *P*1	Melting point: 484 K
*a* = 9.3438 (5) Å	Mo *K*α radiation, λ = 0.71073 Å
*b* = 10.0736 (5) Å	Cell parameters from 3249 reflections
*c* = 10.8452 (5) Å	θ = 3.9–26.3°
α = 86.832 (4)°	µ = 2.10 mm^−^^1^
β = 77.203 (4)°	*T* = 200 K
γ = 84.674 (4)°	Platelet, yellow
*V* = 990.53 (9) Å^3^	0.28 × 0.24 × 0.05 mm
*Z* = 2	

### Data collection

**Table:**

Oxford XCalibur diffractometer	4004 independent reflections
Radiation source: fine-focus sealed tube	2664 reflections with *I* > 2σ(*I*)
graphite	*R*_int_ = 0.030
Detector resolution: 15.9809 pixels mm^-1^	θ_max_ = 26.3°, θ_min_ = 3.9°
ω scans	*h* = −11→11
Absorption correction: multi-scan (*CrysAlis RED*; Oxford Diffraction, 2006)	*k* = −12→12
*T*_min_ = 0.783, *T*_max_ = 1.000	*l* = −13→12
7695 measured reflections	

### Refinement

**Table:**

Refinement on *F*^2^	Primary atom site location: structure-invariant direct methods
Least-squares matrix: full	Secondary atom site location: difference Fourier map
*R*[*F*^2^ > 2σ(*F*^2^)] = 0.034	Hydrogen site location: inferred from neighbouring sites
*wR*(*F*^2^) = 0.057	H-atom parameters constrained
*S* = 0.84	*w* = 1/[σ^2^(*F*_o_^2^) + (0.0232*P*)^2^] where *P* = (*F*_o_^2^ + 2*F*_c_^2^)/3
4004 reflections	(Δ/σ)_max_ = 0.001
256 parameters	Δρ_max_ = 0.60 e Å^−^^3^
0 restraints	Δρ_min_ = −0.35 e Å^−^^3^

### Special details

**Table:**

Experimental. CrysAlis RED, Oxford Diffraction Ltd., Version 1.171.32.29 (release 10-06-2008 CrysAlis171 .NET) (compiled Jun 10 2008,16:49:55) Empirical absorption correction using spherical harmonics, implemented in SCALE3 ABSPACK scaling algorithm.

### Fractional atomic coordinates and isotropic or equivalent isotropic displacement parameters (Å^2^)

**Table:**

	*x*	*y*	*z*	*U*_iso_*/*U*_eq_	
O1	0.29606 (17)	0.74651 (15)	0.40530 (15)	0.0371 (4)	
H1	0.2997	0.7026	0.4728	0.056*	
N1	0.14604 (19)	0.98882 (17)	0.28625 (17)	0.0320 (5)	
H71	0.0504	1.0043	0.2930	0.038*	
N2	0.38107 (18)	0.90626 (17)	0.17422 (16)	0.0253 (4)	
N3	0.59036 (18)	0.96092 (17)	0.20465 (17)	0.0281 (5)	
H73	0.6551	0.9964	0.2378	0.034*	
C1	0.2175 (2)	0.6833 (2)	0.3382 (2)	0.0261 (5)	
C2	0.1706 (2)	0.5575 (2)	0.3723 (2)	0.0314 (6)	
H2A	0.1938	0.5116	0.4452	0.038*	
C3	0.0906 (2)	0.4986 (2)	0.3012 (2)	0.0388 (6)	
H3	0.0573	0.4129	0.3264	0.047*	
C4	0.0583 (3)	0.5626 (2)	0.1941 (2)	0.0394 (6)	
H4	0.0040	0.5211	0.1447	0.047*	
C5	0.1056 (2)	0.6874 (2)	0.1593 (2)	0.0324 (6)	
H5	0.0842	0.7313	0.0848	0.039*	
C6	0.1839 (2)	0.7507 (2)	0.2306 (2)	0.0243 (5)	
C7	0.2219 (2)	0.8922 (2)	0.1944 (2)	0.0248 (5)	
H7	0.1918	0.9171	0.1127	0.030*	
C8	0.2073 (2)	1.0582 (2)	0.3632 (2)	0.0268 (5)	
C9	0.1194 (2)	1.1389 (2)	0.4568 (2)	0.0339 (6)	
H9	0.0153	1.1454	0.4678	0.041*	
C10	0.1830 (3)	1.2084 (2)	0.5323 (2)	0.0393 (6)	
H10	0.1218	1.2622	0.5957	0.047*	
C11	0.3347 (3)	1.2023 (2)	0.5187 (2)	0.0369 (6)	
H11	0.3769	1.2511	0.5721	0.044*	
C12	0.4231 (3)	1.1247 (2)	0.4269 (2)	0.0329 (6)	
H12	0.5270	1.1201	0.4163	0.039*	
C13	0.3605 (2)	1.0524 (2)	0.3490 (2)	0.0251 (5)	
C14	0.4443 (2)	0.9756 (2)	0.2468 (2)	0.0249 (5)	
C15	0.4906 (2)	0.8449 (2)	0.0805 (2)	0.0234 (5)	
C16	0.6239 (2)	0.8808 (2)	0.1001 (2)	0.0248 (5)	
C17	0.7569 (2)	0.8389 (2)	0.0233 (2)	0.0300 (6)	
H17	0.8475	0.8646	0.0364	0.036*	
C18	0.7518 (2)	0.7580 (2)	−0.0733 (2)	0.0337 (6)	
H18	0.8411	0.7264	−0.1280	0.040*	
C19	0.6187 (3)	0.7212 (2)	−0.0929 (2)	0.0337 (6)	
H19	0.6199	0.6650	−0.1607	0.040*	
C20	0.4854 (2)	0.7640 (2)	−0.0169 (2)	0.0315 (6)	
H20	0.3948	0.7391	−0.0307	0.038*	
O2	0.7042 (2)	0.38247 (17)	0.37419 (15)	0.0512 (5)	
H2	0.7545	0.3286	0.3219	0.077*	
C21	0.5862 (3)	0.4457 (3)	0.3245 (3)	0.0556 (8)	
H21A	0.5252	0.5062	0.3880	0.067*	
H21B	0.5236	0.3771	0.3092	0.067*	
C22	0.6360 (3)	0.5233 (3)	0.2049 (3)	0.0656 (9)	
H22A	0.7003	0.5901	0.2188	0.098*	
H22B	0.5503	0.5683	0.1774	0.098*	
H22C	0.6905	0.4630	0.1396	0.098*	
Br1	0.85598 (3)	0.14400 (3)	0.18829 (3)	0.04163 (10)	

### Atomic displacement parameters (Å^2^)

**Table:**

	*U*^11^	*U*^22^	*U*^33^	*U*^12^	*U*^13^	*U*^23^
O1	0.0462 (10)	0.0364 (10)	0.0337 (11)	−0.0104 (8)	−0.0197 (9)	0.0112 (8)
N1	0.0167 (10)	0.0352 (12)	0.0412 (13)	0.0017 (9)	−0.0023 (9)	0.0008 (10)
N2	0.0197 (10)	0.0283 (11)	0.0270 (12)	−0.0017 (8)	−0.0045 (9)	0.0048 (9)
N3	0.0191 (10)	0.0346 (12)	0.0317 (13)	−0.0045 (8)	−0.0079 (9)	0.0041 (9)
C1	0.0221 (12)	0.0280 (14)	0.0274 (15)	−0.0020 (10)	−0.0036 (11)	−0.0022 (11)
C2	0.0353 (14)	0.0265 (14)	0.0296 (16)	0.0007 (11)	−0.0032 (12)	0.0029 (11)
C3	0.0381 (15)	0.0302 (15)	0.0441 (18)	−0.0069 (12)	0.0013 (13)	−0.0007 (13)
C4	0.0369 (15)	0.0384 (16)	0.0452 (19)	−0.0079 (12)	−0.0097 (13)	−0.0103 (13)
C5	0.0271 (13)	0.0407 (16)	0.0291 (15)	−0.0014 (12)	−0.0064 (11)	0.0007 (12)
C6	0.0170 (12)	0.0280 (13)	0.0257 (15)	−0.0013 (10)	−0.0007 (10)	0.0020 (11)
C7	0.0168 (12)	0.0326 (14)	0.0238 (14)	−0.0003 (10)	−0.0044 (10)	0.0065 (11)
C8	0.0299 (14)	0.0212 (13)	0.0265 (15)	−0.0022 (11)	−0.0027 (11)	0.0084 (11)
C9	0.0248 (13)	0.0294 (14)	0.0409 (17)	0.0003 (11)	0.0037 (12)	0.0067 (12)
C10	0.0484 (17)	0.0286 (15)	0.0329 (16)	0.0009 (12)	0.0072 (13)	−0.0028 (12)
C11	0.0421 (16)	0.0345 (15)	0.0332 (17)	−0.0057 (12)	−0.0056 (13)	−0.0005 (12)
C12	0.0300 (14)	0.0313 (15)	0.0363 (16)	−0.0045 (11)	−0.0060 (12)	0.0068 (12)
C13	0.0253 (13)	0.0246 (13)	0.0221 (14)	−0.0006 (10)	−0.0002 (10)	0.0052 (11)
C14	0.0201 (13)	0.0257 (13)	0.0269 (15)	−0.0018 (10)	−0.0034 (11)	0.0093 (11)
C15	0.0214 (12)	0.0229 (13)	0.0226 (14)	0.0005 (10)	0.0000 (10)	0.0055 (10)
C16	0.0268 (13)	0.0240 (13)	0.0224 (15)	−0.0012 (10)	−0.0044 (11)	0.0052 (11)
C17	0.0221 (13)	0.0276 (14)	0.0382 (16)	0.0000 (10)	−0.0044 (11)	0.0055 (12)
C18	0.0287 (14)	0.0324 (15)	0.0333 (16)	0.0033 (11)	0.0041 (11)	0.0043 (12)
C19	0.0407 (16)	0.0338 (15)	0.0255 (15)	−0.0018 (12)	−0.0043 (12)	−0.0042 (11)
C20	0.0258 (14)	0.0369 (15)	0.0329 (16)	−0.0051 (11)	−0.0096 (12)	0.0059 (12)
O2	0.0656 (13)	0.0575 (13)	0.0339 (12)	−0.0002 (10)	−0.0217 (10)	0.0040 (9)
C21	0.064 (2)	0.0533 (19)	0.050 (2)	−0.0028 (15)	−0.0179 (16)	0.0123 (16)
C22	0.086 (2)	0.0533 (19)	0.060 (2)	−0.0077 (17)	−0.0255 (18)	0.0227 (17)
Br1	0.02457 (14)	0.04927 (18)	0.0522 (2)	−0.00537 (11)	−0.01119 (12)	0.00401 (13)

### Geometric parameters (Å, °)

**Table:**

O1—C1	1.360 (2)	C9—H9	0.9500
O1—H1	0.8400	C10—C11	1.388 (3)
N1—C8	1.365 (3)	C10—H10	0.9500
N1—C7	1.452 (3)	C11—C12	1.373 (3)
N1—H71	0.8800	C11—H11	0.9500
N2—C14	1.342 (3)	C12—C13	1.397 (3)
N2—C15	1.398 (3)	C12—H12	0.9500
N2—C7	1.474 (2)	C13—C14	1.427 (3)
N3—C14	1.336 (2)	C15—C20	1.381 (3)
N3—C16	1.390 (3)	C15—C16	1.392 (3)
N3—H73	0.8800	C16—C17	1.379 (3)
C1—C2	1.383 (3)	C17—C18	1.373 (3)
C1—C6	1.398 (3)	C17—H17	0.9500
C2—C3	1.376 (3)	C18—C19	1.393 (3)
C2—H2A	0.9500	C18—H18	0.9500
C3—C4	1.376 (3)	C19—C20	1.380 (3)
C3—H3	0.9500	C19—H19	0.9500
C4—C5	1.377 (3)	C20—H20	0.9500
C4—H4	0.9500	O2—C21	1.416 (3)
C5—C6	1.390 (3)	O2—H2	0.8400
C5—H5	0.9500	C21—C22	1.482 (3)
C6—C7	1.511 (3)	C21—H21A	0.9900
C7—H7	1.0000	C21—H21B	0.9900
C8—C9	1.401 (3)	C22—H22A	0.9800
C8—C13	1.402 (3)	C22—H22B	0.9800
C9—C10	1.367 (3)	C22—H22C	0.9800
			
?···?	?		
			
C1—O1—H1	109.5	C12—C11—C10	119.2 (2)
C8—N1—C7	126.93 (17)	C12—C11—H11	120.4
C8—N1—H71	116.5	C10—C11—H11	120.4
C7—N1—H71	116.5	C11—C12—C13	120.2 (2)
C14—N2—C15	109.14 (17)	C11—C12—H12	119.9
C14—N2—C7	125.15 (18)	C13—C12—H12	119.9
C15—N2—C7	125.64 (18)	C12—C13—C8	120.6 (2)
C14—N3—C16	109.31 (18)	C12—C13—C14	123.8 (2)
C14—N3—H73	125.3	C8—C13—C14	115.6 (2)
C16—N3—H73	125.3	N3—C14—N2	108.79 (19)
O1—C1—C2	122.8 (2)	N3—C14—C13	128.8 (2)
O1—C1—C6	117.34 (19)	N2—C14—C13	122.39 (19)
C2—C1—C6	119.9 (2)	C20—C15—C16	121.4 (2)
C3—C2—C1	120.3 (2)	C20—C15—N2	132.57 (19)
C3—C2—H2A	119.8	C16—C15—N2	106.04 (19)
C1—C2—H2A	119.8	C17—C16—N3	131.3 (2)
C2—C3—C4	120.7 (2)	C17—C16—C15	122.0 (2)
C2—C3—H3	119.7	N3—C16—C15	106.71 (19)
C4—C3—H3	119.7	C18—C17—C16	116.6 (2)
C3—C4—C5	119.2 (2)	C18—C17—H17	121.7
C3—C4—H4	120.4	C16—C17—H17	121.7
C5—C4—H4	120.4	C17—C18—C19	121.6 (2)
C4—C5—C6	121.5 (2)	C17—C18—H18	119.2
C4—C5—H5	119.3	C19—C18—H18	119.2
C6—C5—H5	119.3	C20—C19—C18	121.9 (2)
C5—C6—C1	118.4 (2)	C20—C19—H19	119.0
C5—C6—C7	119.27 (19)	C18—C19—H19	119.0
C1—C6—C7	122.17 (19)	C19—C20—C15	116.5 (2)
N1—C7—N2	107.70 (17)	C19—C20—H20	121.8
N1—C7—C6	113.67 (17)	C15—C20—H20	121.8
N2—C7—C6	112.43 (15)	C21—O2—H2	109.5
N1—C7—H7	107.6	O2—C21—C22	113.1 (2)
N2—C7—H7	107.6	O2—C21—H21A	109.0
C6—C7—H7	107.6	C22—C21—H21A	109.0
N1—C8—C9	121.2 (2)	O2—C21—H21B	109.0
N1—C8—C13	120.7 (2)	C22—C21—H21B	109.0
C9—C8—C13	118.2 (2)	H21A—C21—H21B	107.8
C10—C9—C8	120.3 (2)	C21—C22—H22A	109.5
C10—C9—H9	119.9	C21—C22—H22B	109.5
C8—C9—H9	119.9	H22A—C22—H22B	109.5
C9—C10—C11	121.6 (2)	C21—C22—H22C	109.5
C9—C10—H10	119.2	H22A—C22—H22C	109.5
C11—C10—H10	119.2	H22B—C22—H22C	109.5
			
O1—C1—C2—C3	179.6 (2)	C9—C8—C13—C12	0.4 (3)
C6—C1—C2—C3	−0.3 (3)	N1—C8—C13—C14	2.8 (3)
C1—C2—C3—C4	1.2 (3)	C9—C8—C13—C14	−176.06 (19)
C2—C3—C4—C5	−0.8 (3)	C16—N3—C14—N2	0.7 (2)
C3—C4—C5—C6	−0.6 (3)	C16—N3—C14—C13	−177.9 (2)
C4—C5—C6—C1	1.5 (3)	C15—N2—C14—N3	−0.4 (2)
C4—C5—C6—C7	−174.82 (19)	C7—N2—C14—N3	176.86 (17)
O1—C1—C6—C5	179.06 (19)	C15—N2—C14—C13	178.27 (18)
C2—C1—C6—C5	−1.0 (3)	C7—N2—C14—C13	−4.4 (3)
O1—C1—C6—C7	−4.7 (3)	C12—C13—C14—N3	−2.1 (3)
C2—C1—C6—C7	175.17 (19)	C8—C13—C14—N3	174.23 (19)
C8—N1—C7—N2	−14.3 (3)	C12—C13—C14—N2	179.4 (2)
C8—N1—C7—C6	110.9 (2)	C8—C13—C14—N2	−4.2 (3)
C14—N2—C7—N1	12.6 (3)	C14—N2—C15—C20	−179.8 (2)
C15—N2—C7—N1	−170.59 (17)	C7—N2—C15—C20	2.9 (4)
C14—N2—C7—C6	−113.4 (2)	C14—N2—C15—C16	−0.1 (2)
C15—N2—C7—C6	63.4 (2)	C7—N2—C15—C16	−177.31 (17)
C5—C6—C7—N1	112.5 (2)	C14—N3—C16—C17	178.9 (2)
C1—C6—C7—N1	−63.7 (2)	C14—N3—C16—C15	−0.8 (2)
C5—C6—C7—N2	−124.8 (2)	C20—C15—C16—C17	0.6 (3)
C1—C6—C7—N2	59.0 (3)	N2—C15—C16—C17	−179.22 (19)
C7—N1—C8—C9	−173.6 (2)	C20—C15—C16—N3	−179.71 (19)
C7—N1—C8—C13	7.6 (3)	N2—C15—C16—N3	0.5 (2)
N1—C8—C9—C10	−179.5 (2)	N3—C16—C17—C18	179.6 (2)
C13—C8—C9—C10	−0.7 (3)	C15—C16—C17—C18	−0.8 (3)
C8—C9—C10—C11	0.4 (3)	C16—C17—C18—C19	0.5 (3)
C9—C10—C11—C12	0.1 (3)	C17—C18—C19—C20	0.1 (3)
C10—C11—C12—C13	−0.4 (3)	C18—C19—C20—C15	−0.3 (3)
C11—C12—C13—C8	0.1 (3)	C16—C15—C20—C19	0.0 (3)
C11—C12—C13—C14	176.3 (2)	N2—C15—C20—C19	179.8 (2)
N1—C8—C13—C12	179.27 (19)		

### Hydrogen-bond geometry (Å, °)

**Table:**

*D*—H···*A*	*D*—H	H···*A*	*D*···*A*	*D*—H···*A*
O1—H1···O2^i^	0.84	1.82	2.657 (2)	175
N1—H71···Br1^ii^	0.88	2.61	3.3501 (17)	142
N3—H73···Br1^iii^	0.88	2.45	3.1956 (18)	143
O2—H2···Br1	0.84	2.41	3.2378 (17)	168

Symmetry codes: (i) −*x*+1, −*y*+1, −*z*+1; (ii) *x*−1, *y*+1, *z*; (iii) *x*, *y*+1, *z*.
